# Case Report and Systematic Review: Sarcomatoid Parathyroid Carcinoma—A Rare, Highly Malignant Subtype

**DOI:** 10.3389/fendo.2021.793718

**Published:** 2021-12-15

**Authors:** Yongchao Yu, Yue Wang, Qingcheng Wu, Xuzi Zhao, Deshun Liu, Yongfu Zhao, Yuguo Li, Guangzhi Wang, Jingchao Xu, Junzhu Chen, Ning Zhang, Xiaofeng Tian

**Affiliations:** Department of Thyroid Surgery, The Second Hospital of Dalian Medical University, Dalian, China

**Keywords:** sarcomatoid, PTH, parathyroid carcinoma, diagnosis, surgery

## Abstract

**Background:**

Parathyroid carcinoma (PC) is a rare malignancy, the incidence of which is less than 1/1 million per year. Sarcomatoid parathyroid carcinoma (SaPC) is an extremely peculiar subtype; only three cases have been reported internationally. It consists of both malignant epithelial components and sarcomatoid components (mesenchymal origin) simultaneously. This “confusing” cancer exhibits higher invasiveness, and traditional surgery does not appear to achieve the expectation, which differs significantly from that of general PC.

**Objective:**

To characterize the clinicopathologic features of SaPC and explore similarities and differences between SaPC and general PC.

**Materials and Methods:**

We collected clinical data of SaPC cases from our center and literature. The SaPC case in our center was presented. To better understand the characteristics of SaPC, we also reviewed clinical information in general PC cases from our center and literature within the last 5 years, and a systematic review was performed for further comparison.

**Results:**

A 60-year-old woman was admitted for a neck mass and hoarseness. After the surgery, she was confirmed as SaPC and ultimately developed local recurrence at 3 months. Together with the reported cases from literature, four cases of SaPC (three cases from literature) and 203 cases of general PC (200 cases from literature) were reviewed. Both tumors showed obvious abnormalities in parathormone (PTH) level and gland size. Compared to general PC, SaPC has a later age of onset (60.50 ± 7.42 vs. 51.50 ± 8.29), relatively low levels of PTH (110.28 ± 59.32 vs. 1,156.07 ± 858.18), and a larger tumor size (6.00 ± 1.63 vs. 3.14 ± 0.70). For SaPC, all four cases were initially misdiagnosed as thyroid tumors (4/4). Spindle cell areas or transitional zones were common pathological features in SaPC cases (3/4).

**Conclusion:**

SaPC is a very rare pathologic subtype of PC and appears to be much more easily misdiagnosed as a thyroid tumor. Spindle cell areas or transitional zones are highly possible to be pathological features in its sarcomatoid components. Despite many similarities, there are some differences between SaPC and general PC—SaPC does not show the obvious endocrine feature but stronger aggressiveness. Surgical treatment of SaPC does relieve life-threatening symptoms and improve quality of life even with recurrence in the short term.

## Introduction

Parathyroid carcinoma (PC) is a rare malignant neoplasm. The incidence is less than 1 in 1 million per year (1). At present, general PC is believed to be a slowly progressive disease characterized by specific clinical features (2), and radical surgery is believed to be the only effective curative treatment for this disease (3–5). Sarcomatoid carcinoma is an exceedingly rare tumor subtype. It refers to a bipolar malignancy with both carcinoma and sarcomatoid components. In PC, this tumor subtype was initially named parathyroid carcinosarcoma or parathyroid carcinoma with sarcomatoid differentiation (6–8). We utilized the term “sarcomatoid carcinoma” referring to nomenclature of other tumors with similar features. Sarcomatoid parathyroid carcinoma (SaPC) was first reported in 2002 by Nacamuli et al. (6) and characterized by strong invasiveness and life-threatening recurrence shortly after the operation, which is quite different from general PC. For sarcomatoid carcinoma, there are several hypotheses about its formation, and epithelial–mesenchymal transformation (EMT) appears to play a major role (9, 10).

Here, we introduce similarities and differences between SaPC and general PC based on our experience with cases in our center and the literature. In addition, we provide more insights into the diagnosis and surgical strategies of SaPC.

## Materials and Methods

### The Collection of Medical Records

From January 2016 to September 2021, four patients with PC were enrolled in this study, including one patient with SaPC (one female) and three patients with general PC (three females). They underwent surgery in our department and were followed up. We obtained clinicopathologic and prognostic information of the patients after approval by the Ethics Committee of the Second Hospital of Dalian Medical University.

### Systematic Review

The systematic review was conducted under the Preferred Reporting Items for Systematic reviews and Meta-Analyses (PRISMA) guidelines (11). Literature that contained general PC cases was searched using terms “parathyroid carcinoma” OR “parathyroid cancer” in PubMed (MeSH) and Cochrane Library (Title Abstract Keyword) from 2016 to present. General PC cases were defined as an age of onset >18 years old, having elevated preoperative parathormone (PTH) level (>65 pg/ml), and finally diagnosed with PC (based on postoperative histopathology). Literature related to SaPC was obtained by manual searches. Only publications in English were considered. The date of the last retrieval was September 18, 2021, and literature was reviewed independently by two authors. For all enrolled cases, we reviewed age, gender, preoperative total serum calcium level, preoperative PTH, and tumor size (the greatest diameter) for further analysis. Prognostic information is also reviewed in SaPC cases. SPSS software 23.0 is used for statistical analyses, and GraphPad Prism 8.0 software is used for data plotting. Data are expressed as mean ± SD. Two-tailed independent samples t-test is used for evaluating statistical differences; *p* < 0.05 is considered significant (*), and *p* < 0.01 is considered extremely significant (**).

## Results

### Case Presentation

**Figure 1:**
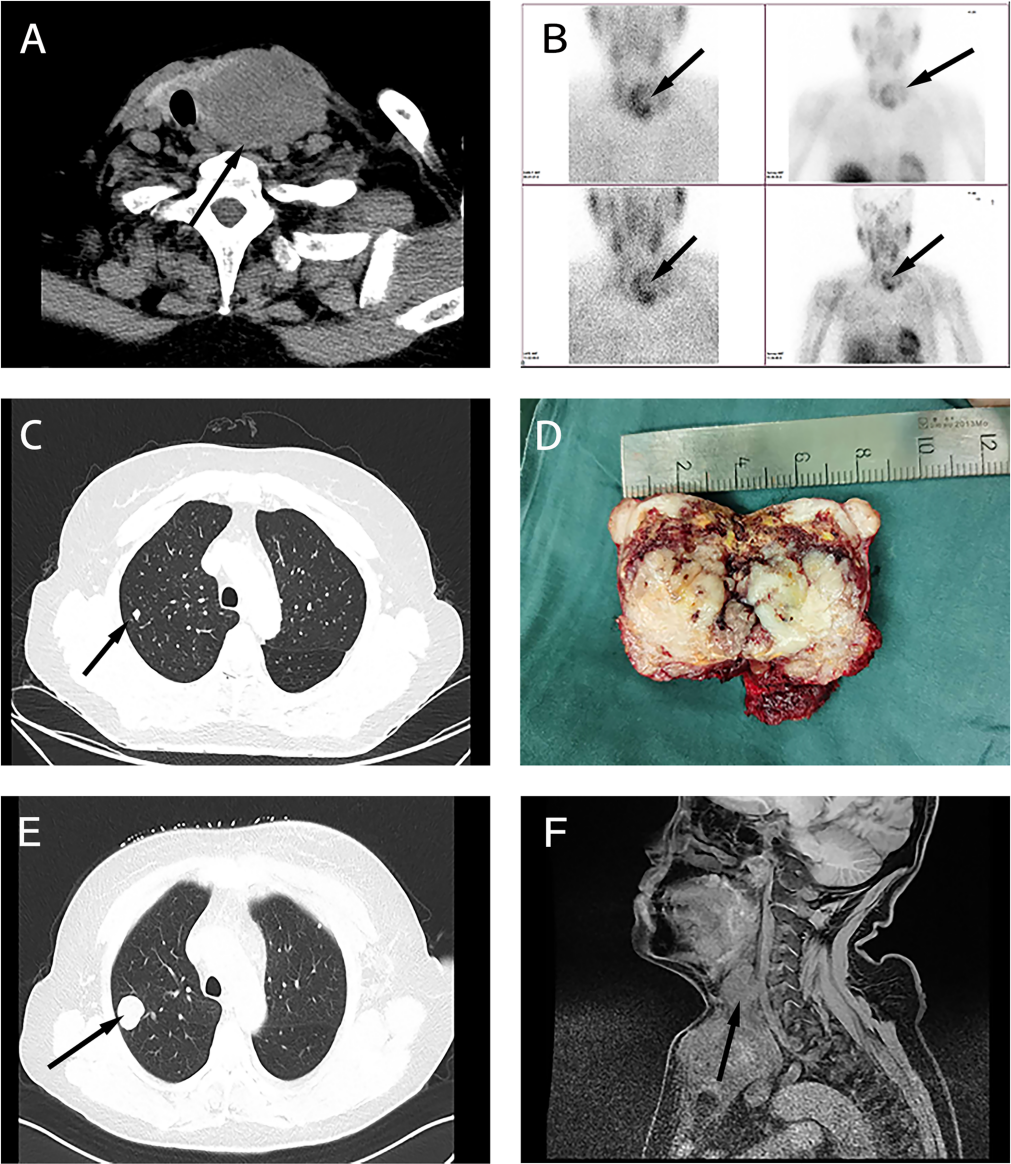
Clinical characteristics of the sarcomatoid parathyroid carcinoma (SaPC) case. **(A)** Neck CT showed the giant tumor. **(B)** Tc-99m sestamibi scintigraphy suggests abnormal nuclide uptake in the left thyroid. **(C)** Chest CT presented micro pulmonary nodule before surgery. **(D)** The profile of the resected tumor. **(E)** Chest CT shows pulmonary metastasis at the closely located position in preoperative chest CT **(C)**. **(F)** Enhanced MRI showed extensive local organ and tissue invasion.

A 60-year-old woman was admitted with a continuously enlarged neck mass for 1 year and hoarseness for 1 week. In addition, she presented with dyspnea for 5 months. The patient had no family history of parathyroid diseases or hyperparathyroidism-jaw tumor syndrome. Physical examination showed a firm left neck mass of approximately 6.0 cm * 5.0 cm. Laboratory findings revealed elevated serum PTH (188.1 pg/ml, reference range: 15–65 pg/ml) and hypercalcemia (total serum calcium: 3.29 mmol/L, reference range: 2.1–2.6 mmol/L). Indicators related to thyroid function were within normal limits. Laryngoscopy showed left vocal cord paralysis. Ultrasonography showed that the left thyroid lobe was enlarged significantly, a hypoechoic lesion nearly occupied the whole lobe, and comparable signs were presented on the neck CT (**Figure 1A**). Tc-99m sestamibi scintigraphy demonstrated two-phase nuclide accumulation on the left thyroid (**Figure 1B**). Chest CT showed multiple micro pulmonary nodules (**Figure 1C**).

**Figure 2:**
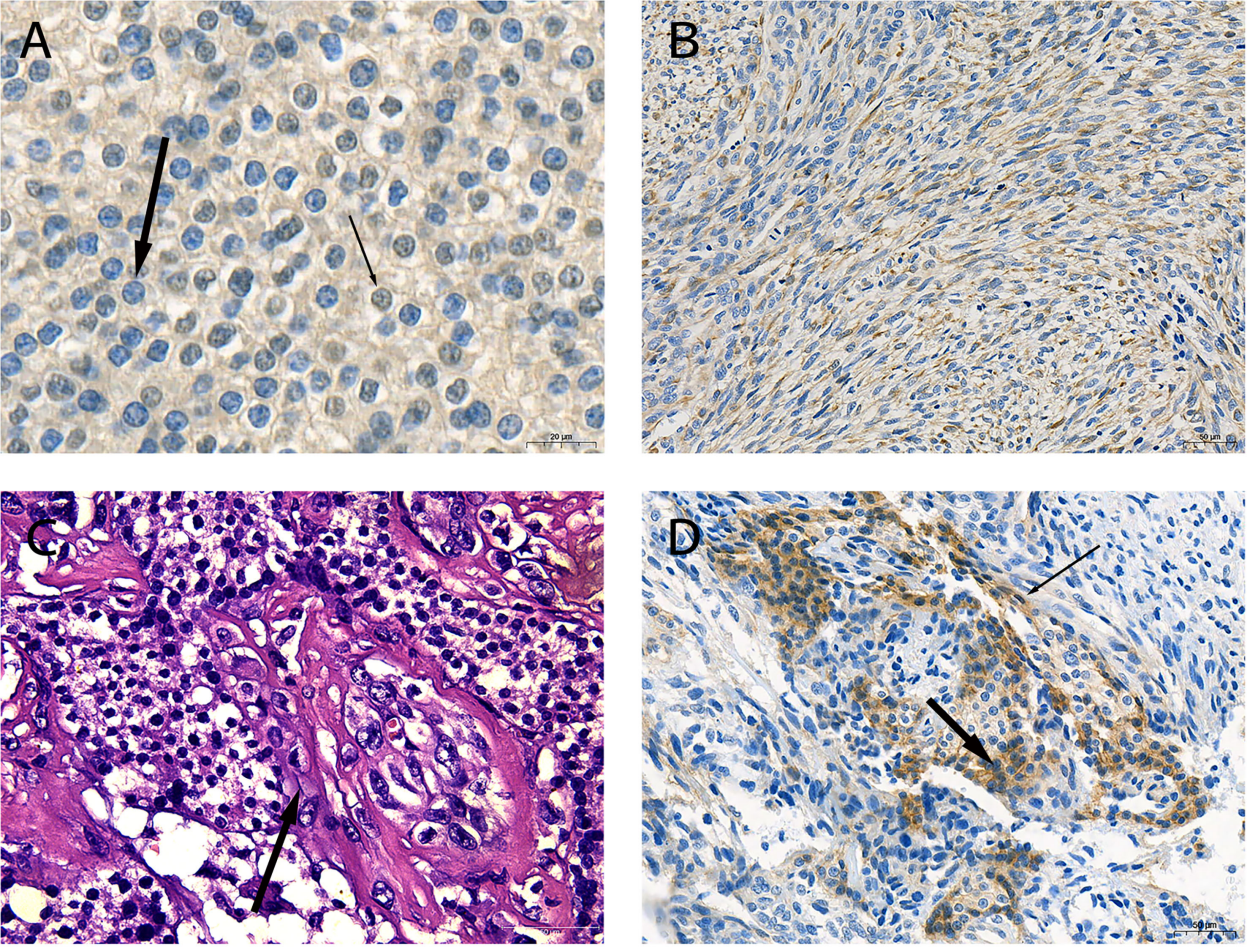
Pathological characteristics of the sarcomatoid parathyroid carcinoma (SaPC) case. **(A)** Negative staining of parafibromin in the nucleus (thick arrow) and contrast-positive staining (thin arrow) of parathyroid carcinoma (PC) cells (×630). **(B)** Spindle cells shuttled through carcinomatous component regions showed positive desmin staining (×200). **(C)** The morphology of cell changes between two component regions (arrow) (H&E, ×400). **(D)** N-cad was positive in carcinomatous (thick arrow) and spindle cell components (thin arrow) (×200).

During the surgical exploration, we found that the tumor invaded the anterior cervical muscle group and left recurrent laryngeal nerve. Only the superior parathyroid was found in the left neck. *En bloc* resection (including part of the invaded recurrent laryngeal nerve and muscle tissue and entire thyroid) and left central lymph node dissection were performed to completely remove the affected tissue. The tumor profile showed that the thyroid was markedly infiltrated, and the normal gland was almost invisible (**Figure 1D**). Postoperative histopathological findings revealed that SaPC widely invaded the ipsilateral thyroid, and 1/6 of the lymph nodes showed metastasis. Immunohistochemical staining was further performed to confirm the diagnosis (**Figure 2**); results were presented below: (1) Carcinomatous components: Some PC cells show negative nuclear staining of parafibromin (**Figure 2A**); Cytokeratin (AE1/AE3) (+); Chromogranin A (+); E-Cadherin (+); PTH (+); Calcitonin (–); Thyroglobulin (-); Desmin (-); KI-67 index 10%; (2) Spindle cell components: Desmin (+; **Figure 2B**); Cytokeratin (AE1/AE3) (-); Chromogranin A (-); E-Cadherin (-); Calcitonin (-); KI-67 index 30%. In addition, the existence of transition zones (**Figure 2C**) and positive N-cad staining in both carcinomatous and sarcomatoid components (**Figure 2D**) was found during pathological examination.

The patient recovered soon postoperatively and remained hoarse. She did not experience choking when drinking water, and dyspnea significantly improved. Three months later, the patient complained of progressively aggravating dyspnea and a gradually growing neck mass. Serum calcium and PTH levels were without abnormal elevation during this time (**Figure S1**). Clinical examinations suggested regional relapse and multiple pulmonary metastases (**Figure 1E**). In contrast to the chest CT before, it seemed that pulmonary metastasis had occurred before the first surgery. Enhanced MRI showed extensive local organ and tissue invasion by the recurred tumor (**Figure 1F**). At last, the patient gave up the medical treatments.

### Systematic Review

**Figure 3:**
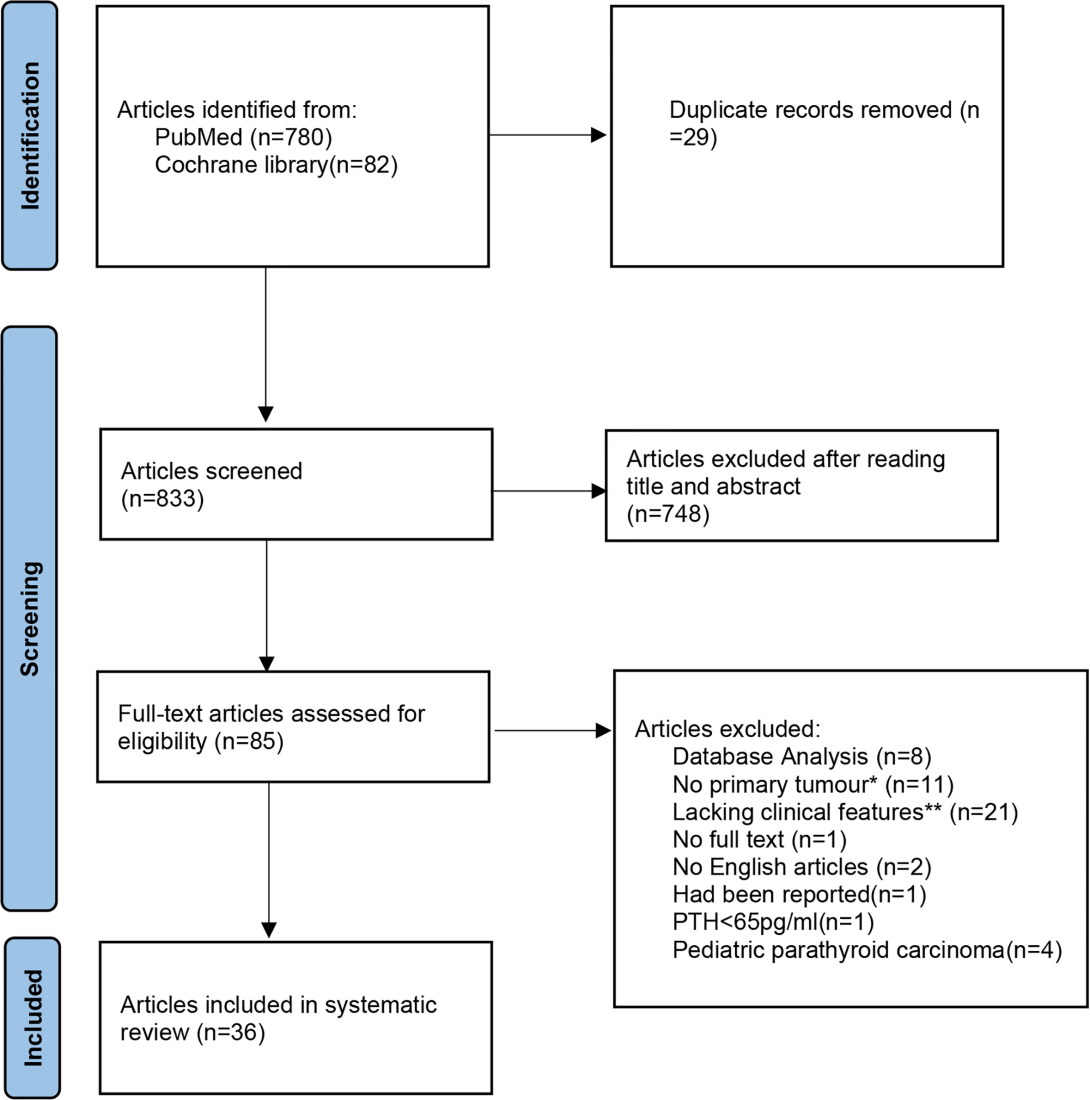
The detailed literature search process presented in a Preferred Reporting Items for Systematic reviews and Meta-Analyses (PRISMA) flowchart. *No primary tumours: secondary to other parathyroid diseases or not identified as parathyroid cancer in the initial surgery. **Lacking clinical features: Articles which lacking more than two study elements in the systematic review (age, gender, preoperative PTH, preoperative total serum calcium or tumour size.

Together with the reported cases, four cases of SaPC (male:female = 1:1, three cases from literature) and 203 cases of general PC (male:female = 0.85:1, 200 cases from literature) were included in this study. The process of the assessment of literature related to general PC was shown in the PRISMA flowchart (**Figure 3**). The details of all four SaPC cases are present in **Table 1**. A total of 197 PTH results, 169 total serum calcium results and 189 tumor size results of general PC cases are reviewed, which are presented in the online supplementary material [**Table S1** (12–47)]. Overall, both SaPC and general PC cases showed abnormal PTH levels and gland size. Compared with general PC, SaPC presented a later age of onset (60.50 ± 7.42 vs. 51.50 ± 8.29, p = 0.032*), lower levels of PTH (110.28 ± 59.32 vs. 1,156.07 ± 858.18, p = 0.016*), and larger tumor size (6.00 ± 1.63 vs. 3.14 ± 0.70, p = 0.039*). Besides, SaPC also shows a lower serum calcium level (2.70 ± 0.42 vs. 3.41 ± 0.91), but it is not significant (p = 0.120) due to small samples. For SaPC cases (**Table 1**), all four cases were initially suspected as thyroid tumors (4/4), three of four patients showed hoarseness (3/4), and two of four patients showed a neck mass (2/4). Spindle cell areas or transitional zones are common pathological features in SaPC cases (3/4).

**Table 1:** Basic clinical data of SaPC cases in our center and literature.

References			Basic information				Laboratory findings		Tumor basic characteristics			Surgery	Postoperative therapy		Prognosis		
Number	Author	Publication date	Sex	Age	Clinical features	Primary diagnosis	PTH (pg/ml)	Ca (mmol/L)	Pathological features	Tumor size (cm)	Local invasion		Radiation therapy	Chemotherapy	Local recurrence	Metastasis	Survival(after the surgery)
No. 1	Our case	2021	Female	60	Left neck mass; hoarseness	Thyroid cancer	188.1	3.29	transitional zone; spindle cell component	6.00	Yes	En bloc resection, Ieft central lymph node dissection	No	No	Yes	Yes	Alive at 6 months
No. 2	Nacamuli (6)	2002	Male	54	Left neck mass	Lymphocytic thyroiditis	117	2.7	transitional zone	8.00	No	En bloc resection	No	Yes	No	Yes	Dead at 7 months
No. 3	Taggart (7)	2013	Female	57	Left neck mass; hoarseness	Thyroid cancer	47	2.45	spindle cell component	4.00	Yes	En bloc resection;cervical lymph node	Yes	Yes	No	Yes	Unknown
No.4	Hu (8)	2020	Male	71	hoarseness	Thyroid cancer	89	2.34	necrosis	6.00	Yes	palliative resection	No	No	Yes	Yes	Dead at 6 months

PTH, parathormone; SaPC, sarcomatoid parathyroid carcinoma.

## Discussion

PC is a rare malignancy in the endocrine system (48). The prevalence of PC in the USA is approximately 30 cases per year (49). For most patients, uncontrolled hypercalcemia is the main cause of death (50). SaPC is a rare and special subtype of PC, and only three cases have been reported internationally. Tumor invasion and metastasis seem to be associated with a patient’s death in SaPC.

Collectively, it is difficult to do the differentiation diagnosis of PC before surgery—how to distinguish malignant from benign, or how to distinguish PC from thyroid tumors, especially in non-functional PC (51) and SaPC. In recent years, the medical circle has made a breakthrough in the management of thyroid cancer, especially with the publication of the 2015 American Thyroid Association (ATA) guidelines (52) in the year 2016. According to the 2015 ATA guidelines, all patients suspected of thyroid cancer need assessment of parathyroid function. Thus, more atypical PC can be found, just like our case. Simultaneously, more cases and clinical experience of PC have been accumulated in recent years. Recognition of parathyroid carcinoma has been enlarged and deepened than any before. Well-recognized clinical manifestations of PC include extremely elevated serum calcium levels and serum PTH levels and a prominent neck mass (53). The significantly increased level of serum alkaline phosphatase has a predictive value for PC (54). Owing to the unclear cytologic atypia between benign and malignant parathyroid tumors, fine-needle aspiration (FNA) results do not diagnose the disease preoperatively instead of increasing the risk of needle tract metastasis (40). Therefore, unlike the thyroid cancer (52), FNA is not applicable when parathyroid diseases are suspected. Imaging examination is routinely used to localize the abnormal parathyroid gland. Ultrasonography and CT are common preoperative examinations before surgery. Methoxy Isobutyl Isonitrile (MIBI) scan is effective in differentiating parathyroid and thyroid tumors when high PTH levels are found in patients. It can also distinguish PC from benign parathyroid diseases by differences in the retention level of 99mTc-MIBI (55). In our study, MIBI scan showed that a large range of abnormal uptake occurred on the left thyroid (**Figure 1B**); this might be a useful adjunct to reveal evidence of invasion when PC is suspected. Definitive diagnosis of PC is restricted to histological findings, which contain observable vascular or peripheral nerve invasion, penetration of the capsule, and infiltration of surrounding tissues (56). Currently, it is believed that parafibromin (encoded by CDC73/HRPT2) is a significant immunohistochemical marker, and there is evidence that its negative expression in parathyroid cells indicates PC (57). Similarly, invasion and negative staining of parafibromin (**Figure 2A**) can be seen in SaPC cases. Recent studies showed that germline CDC73 mutation is closely related to HPT-JT syndrome and other variant phenotypes of sporadic PC (58, 59). There might be a connection between germline CDC73 mutation and SaPC. Unfortunately, we did not conserve the blood sample for the gene test. And the patient was lost to follow-up from December 2020. This is the limitation of this work, and more studies are needed to pursue this connection.

Interestingly, there are some differences in manifestations between SaPC and general PC. Firstly, the endocrine feature of SaPC is less distinct—although the PTH level is higher than normal, it is about one-tenth of general PC, which may be related to functional carcinomatous regions replaced by non-functional sarcomatoid regions at different levels. Moreover, all four cases of SaPC were suspected as thyroid tumors before surgery, which might be associated with the anatomical location and tumor invasiveness, but more cases are needed for validation. Notable signs, such as hoarseness and a larger tumor size, are common in this subtype, which might be an embodiment of the fast disease progression. Pathologically, the diagnosis of SaPC also includes the presence of mesenchymal cell areas and corresponding immunohistochemical markers. Specific spindle cell regions or transitional zones can also be observed in reported cases and our case of SaPC (**Table 1** **;** **Figures 2B, C** **)**, which might be potential histomorphological characteristics of this subtype. In addition, the positive staining of N-cad in both components (**Figure 2D**) and the appearance of transitional zones seem to support the hypothesis of EMT. Combined with our case (**Figure 2B**), sarcomatoid regions showed a positive expression of different mesenchymal tissue markers (desmin or vimentin), indicating that the direction of sarcomatoid differentiation in PC may be different between individuals.

Presently, surgery is the overriding therapeutic modality for PC patients (12, 20, 60). Margin status in the initial surgery is crucial to the prognosis (61), and early salvage surgery can still achieve reasonably good therapeutic efficacy (19). *En bloc* resection with ipsilateral thyroid lobectomy and ipsilateral central lymph node dissection seems to be an appropriate surgical approach (62). However, a study suggested that resection of the ipsilateral thyroid cannot prolong survival (63). Thus, in-depth studies on the extent of resection are needed.

Nevertheless, the effect and extent of surgery in SaPC require further study. In our case (No. 1, **Table 1**), we performed the radical surgical procedure, and dyspnea improved temporarily, but tumor recurrence occurred 3 months later. Combined with reported cases, SaPC showed early recurrence after radical surgery. Surgical treatments in SaPC indeed achieve improvements in clinical symptoms, but recurrence and death occurred about 6 months later. In general, surgery for SaPC does relieve tumor compression, correct hypercalcemia, and improve quality of life even with recurrence in the short term.

According to the literature, radiotherapy and chemotherapy after surgery for general PC are ineffective (64, 65). The result shows no difference in SaPC with these treatments—the first two patients received postoperative chemotherapy but relapsed in months. In terms of sarcomatoid carcinoma in other organs such as kidney (66) and lung (67), they show the similar clinicopathological characteristics as SaPC, and targeted therapy has been confirmed to be potentially beneficial. This may be the future direction of treatment for SaPC.

Due to the rarity of the disease, there are currently no well-accepted staging criteria for PC. Patients who receive radical resection often have a favorable outcome, with a 5-year survival of up to 82.3% and 66% in 10 years (68). In our center, all four patients with typical PC received radical surgery, and none experienced recurrence (follow-up period was between 15 and 66 months; data not shown). However, SaPC was highly aggressive, with postoperative recurrence and early systemic metastases in all four patients (**Table 1**), which led to the patient’s death in a short time—two of the four patients with SaPC died within 7 months. We noticed that a longer period of the systematic review might benefit the research, which is a limitation of this work. We will continue to concern relative research henceforth.

## Conclusion

SaPC is a highly unusual subtype of PC, and patients eventually succumb to direct tumor invasion and metastasis, while general PC has a good prognosis after radical surgery. Patients with SaPC seem to be easily misdiagnosed as thyroid tumors. Spindle cell areas or transitional zones are likely to be pathological features of SaPC. Despite many similarities, there are also some differences between SaPC and general PC—SaPC does not present a significant endocrinological feature but presents more aggressiveness. Surgery in SaPC cases indeed improves severe symptoms and quality of life even in patients experiencing a relapse in the short term, and other effective treatments are needed to explore.

## Data Availability Statement

The original contributions presented in the study are included in the article/**Supplementary Material**. Further inquiries can be directed to the corresponding authors.

## Ethics Statement

The studies involving human participants were reviewed and approved by the Ethics Committee of the Second Hospital of Dalian Medical University. The patients/participants provided their written informed consent to participate in this study.

## Author Contributions

YY designed the study. YY, YW, QW, and XZ reviewed the literature and collected data. DL, GW, JX, and JC analyzed data and assessed the accuracy of results. YY wrote the first paper draft of the article. YL and YZ provided assistance during the writing process. XT and NZ reviewed and edited the article. All authors contributed to the article and approved the submitted version.

## Funding

This study was supported by the Scientific Research Fund of Liaoning Provincial Education Department, China (grant no. LZ2019057).

## Conflict of Interest

The authors declare that the research was conducted in the absence of any commercial or financial relationships that could be construed as a potential conflict of interest.

## Publisher’s Note

All claims expressed in this article are solely those of the authors and do not necessarily represent those of their affiliated organizations, or those of the publisher, the editors and the reviewers. Any product that may be evaluated in this article, or claim that may be made by its manufacturer, is not guaranteed or endorsed by the publisher.
