# Supplementary figures and images for: Case Report and Systematic Review: Sarcomatoid Parathyroid Carcinoma—A Rare, Highly Malignant Subtype
Source: Front Endocrinol (Lausanne). 2021 Dec 15;12:793718. doi: 10.3389/fendo.2021.793718 (PMC8719313; doi:10.3389/fendo.2021.793718)

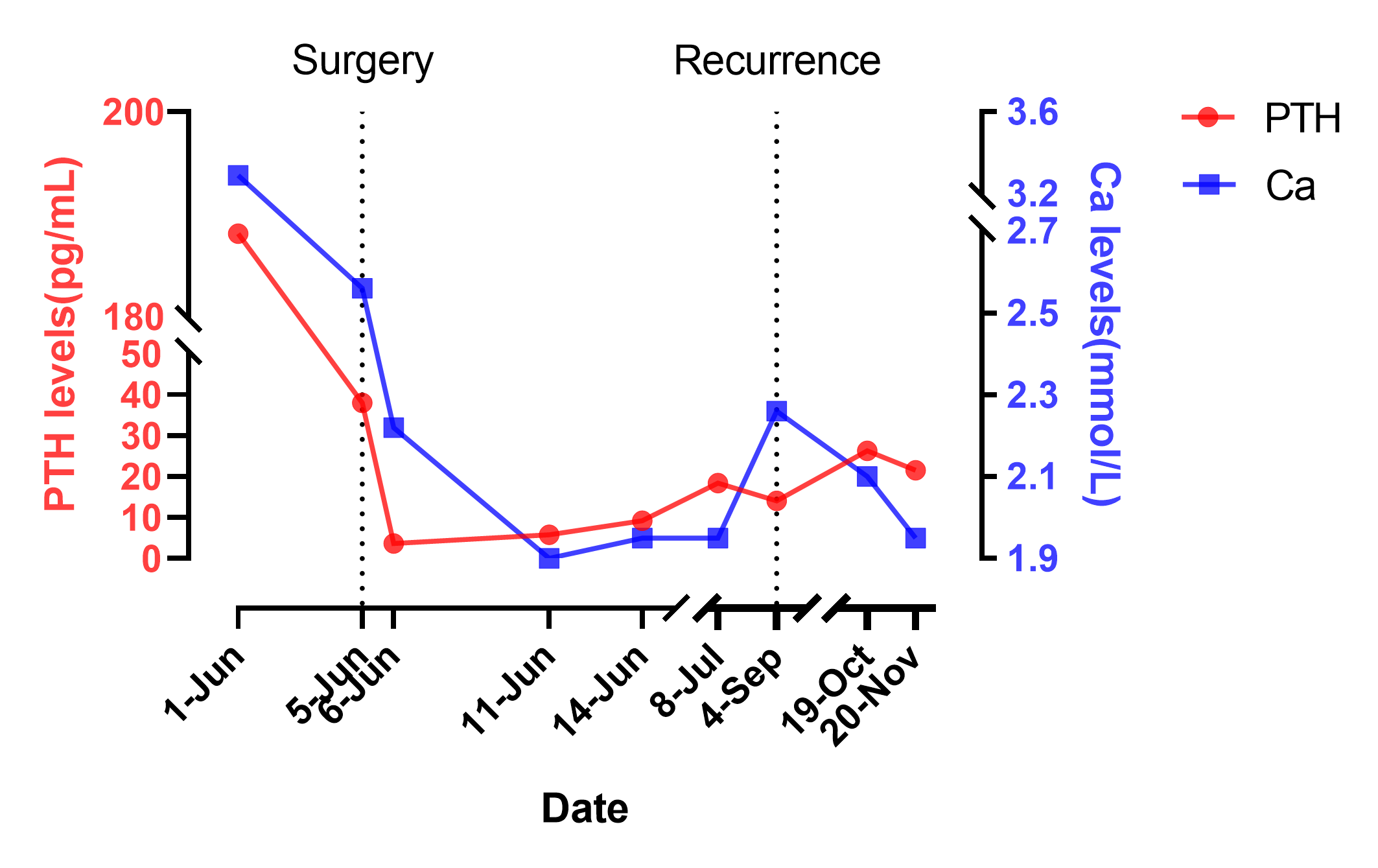

Supplement: Supplementary file 1 [file Image_1.tif]
